# Source-specific nitrate and nitrite intakes and associations with sociodemographic factors in the Danish Diet Cancer and Health cohort

**DOI:** 10.3389/fnut.2024.1326991

**Published:** 2024-02-27

**Authors:** Dorit W. Erichsen, Pratik Pokharel, Cecilie Kyrø, Jörg Schullehner, Liezhou Zhong, Catherine P. Bondonno, Frederik Dalgaard, Peter Fjeldstad Hendriksen, Torben Sigsgaard, Jonathan M. Hodgson, Anja Olsen, Anne Tjønneland, Nicola P. Bondonno

**Affiliations:** ^1^Danish Cancer Institute, Copenhagen, Denmark; ^2^Nutrition and Health Innovation Research Institute, School of Medical and Health Sciences, Edith Cowan University, Perth, WA, Australia; ^3^Department of Groundwater and Quaternary Geology Mapping, Geological Survey of Denmark and Greenland, Aarhus, Denmark; ^4^Department of Public Health, Aarhus University, Aarhus, Denmark; ^5^Medical School, The University of Western Australia, Royal Perth Hospital, Perth, WA, Australia; ^6^Department of Cardiology, Herlev and Gentofte University Hospital, Copenhagen, Denmark; ^7^Danish Big Data Centre for Environment and Health (BERTHA), Aarhus University, Aarhus, Denmark; ^8^Department of Public Health, Faculty of Health and Medical Sciences, University of Copenhagen, Copenhagen, Denmark

**Keywords:** nitrate, nitrite, vegetables, meat, water

## Abstract

**Background:**

The dietary source and intake levels of nitrate and nitrite may govern its deleterious versus beneficial effects on human health. Existing evidence on detailed source-specific intake is limited. The objectives of this study were to assess nitrate and nitrite intakes from different dietary sources (plant-based foods, animal-based foods, and water), characterize the background diets of participants with low and high intakes, and investigate how sociodemographic and lifestyle factors associate with intake levels.

**Methods:**

In the Danish Diet, Cancer and Health Cohort, sociodemographic and lifestyle information was obtained from participants at enrolment (1993–1997). Source-dependent nitrate and nitrite intakes were calculated using comprehensive food composition databases, with tap water nitrate intakes estimated via the national drinking water quality monitoring database linked with participants’ residential addresses from 1978 to 2016. Underlying dietary patterns were examined using radar plots comparing high to low consumers while sociodemographic predictors of source-dependent nitrate intakes were investigated using linear regression models.

**Results:**

In a Danish cohort of 55,754 participants aged 50–65 at enrolment, the median [IQR] intakes of dietary nitrate and nitrite were 58.13 [44.27–74.90] mg/d and 1.79 [1.43–2.21] mg/d, respectively. Plant-based foods accounted for ~76% of nitrate intake, animal-based foods ~10%, and water ~5%. Nitrite intake was sourced roughly equally from plants and animals. Higher plant-sourced nitrate intake was associated with healthier lifestyles, better dietary patterns, more physical activity, higher education, lower age and lower BMI. Females and participants who had never smoked also had significantly higher plant-sourced nitrate intakes. Higher water-sourced nitrate intake was linked to sociodemographic risk factors (smoking, obesity, lower education). Patterns for animal-sourced nitrate were less clear.

**Conclusion:**

Participants with higher plant-sourced nitrate intakes tend to be healthier while participants with higher water-sourced nitrate intakes tended to be unhealthier than their low consuming counterparts. Future research in this cohort should account for the sociodemographic and dietary predictors of source-specific nitrate intake we have identified.

## 1 Introduction

Nitrate and nitrite are controversial dietary components, and the health consequences of their consumption has been a topic of continued debate and research interest. We are continually exposed to nitrate and nitrite, as they are not only formed endogenously but are ubiquitous in nature being found in plants, animal-sourced foods, and water (1). In plants, nitrate and nitrite hold pivotal functions, supporting growth and development (2, 3). There is a wide variation in the nitrate and nitrite content of plants, being influenced by genetic, environmental and cultivation factors (4, 5). In animal-sourced food products, nitrate and nitrite are both naturally occurring (6) and permitted food additives most commonly in the processing of meats (7, 8). The nitrate and nitrite content of both plant-and animal-sourced foods is influenced by cooking (5, 9). Drinking water is a source of nitrate being found naturally in both surface and groundwater due to drainage from soil. Within the soil it constitutes a vital element in the nitrogen cycle. Nitrifying bacteria generate nitrate from nitrogen and ammonia sources. However, the intensification of ammonia-rich fertilizers, wastewater treatment, septic tanks, nitrogen-fixing crop cultivation, and fossil fuel combustion has led to a twofold escalation in nitrate deposition in land, subsequently causing increased nitrate concentrations in water (10).

Nitrate itself is innocuous and evidence points to beneficial physiological effects of plant-sourced nitrate consumption owing to its conversion to nitric oxide (NO) through the enterosalivary nitrate-nitrite-NO pathway (11). These effects include, but are not limited to, a lowering of blood pressure, improved blood vessel function, enhanced exercise performance, tissue protection during ischemia–reperfusion, advanced mitochondrial function, and potential neuroprotective roles (1). However, there is also evidence of possible carcinogenic potential. This worry emerges from the reduction of nitrate to nitrite and subsequent possibility of nitrosation, forming carcinogenic *N*-nitroso compounds (12, 13). In the presence of co-occurring factors found in whole food matrices, the formation of *N*-nitroso compounds from nitrate, after its reduction to nitrite, may be augmented (e.g., in meat owing to the presence of amines, amides or heme iron (14, 15)) while in others it may be inhibited (e.g., in vegetables owing to the presence of antioxidant vitamins and polyphenols (16)). Notably, the carcinogenic properties of *N*-nitroso compounds, stemming from nitrite additives in processed meat, are primary suspects behind the latter’s adverse health impacts (17). As global public health agencies are re-evaluating the use of nitrites and nitrates as food additives (18), a distinction should be made between naturally occurring and additive nitrate/nitrite to provide the evidence needed to guide decision making. Thus, to reach conclusive insights on the health implications of nitrate/nitrite ingestion via drinking water and food, studies must differentiate between nitrate/nitrite sources in the diet, categorize them as inherent or added, and accurately account for individual factors like smoking and dietary habits that can influence endogenous nitrosation (16).

The aim of the present study, conducted in the Danish Diet Cancer and Health cohort, was three-fold: (1) to estimate and describe nitrate/nitrite intakes according to dietary sources in a large cohort of adults—nitrate intake estimates, which have been published previously (19), were updated following the release of more comprehensive nitrate content of food databases (6, 20)—(2) to characterize the background diets of cohort participants with low and high nitrate/nitrite intakes, and (3) to investigate if and how sociodemographic and lifestyle factors were associated with nitrate/nitrite intakes. These findings will inform future studies in this cohort that aim to examine associations between source-dependent nitrate and nitrite intake and various health outcomes.

## 2 Materials and methods

### 2.1 Study population

This study uses data from the Danish Diet, Cancer, and Health (DCH) cohort, for which 57,053 participants without a registered cancer diagnosis, aged 50–65 years and residing in the greater areas of Copenhagen and Aarhus in Denmark, were enrolled between 1993 and 1997. Detailed information on the purpose and design of the cohort have been published elsewhere (21). Briefly, at enrolment, information about diet and lifestyle was obtained by validated, self-administered questionnaires, and anthropometrics were measured by trained personnel during a visit to one of the study centers. For the present study, we excluded all participants with missing dietary data (*n* = 55), missing information on the nitrate content of the waterworks that supplied their place of residence (*n* = 423), or missing or implausible covariate data (*n* = 237), leaving 55,754 participants for analysis (Supplementary Figure 1).

Establishment of the cohort was approved by relevant scientific ethics committees and the Danish Data Protection Agency. All participants gave written informed consent.

### 2.2 Assessment of nitrate and nitrite intake from diet and tap water

**Figure 1:**
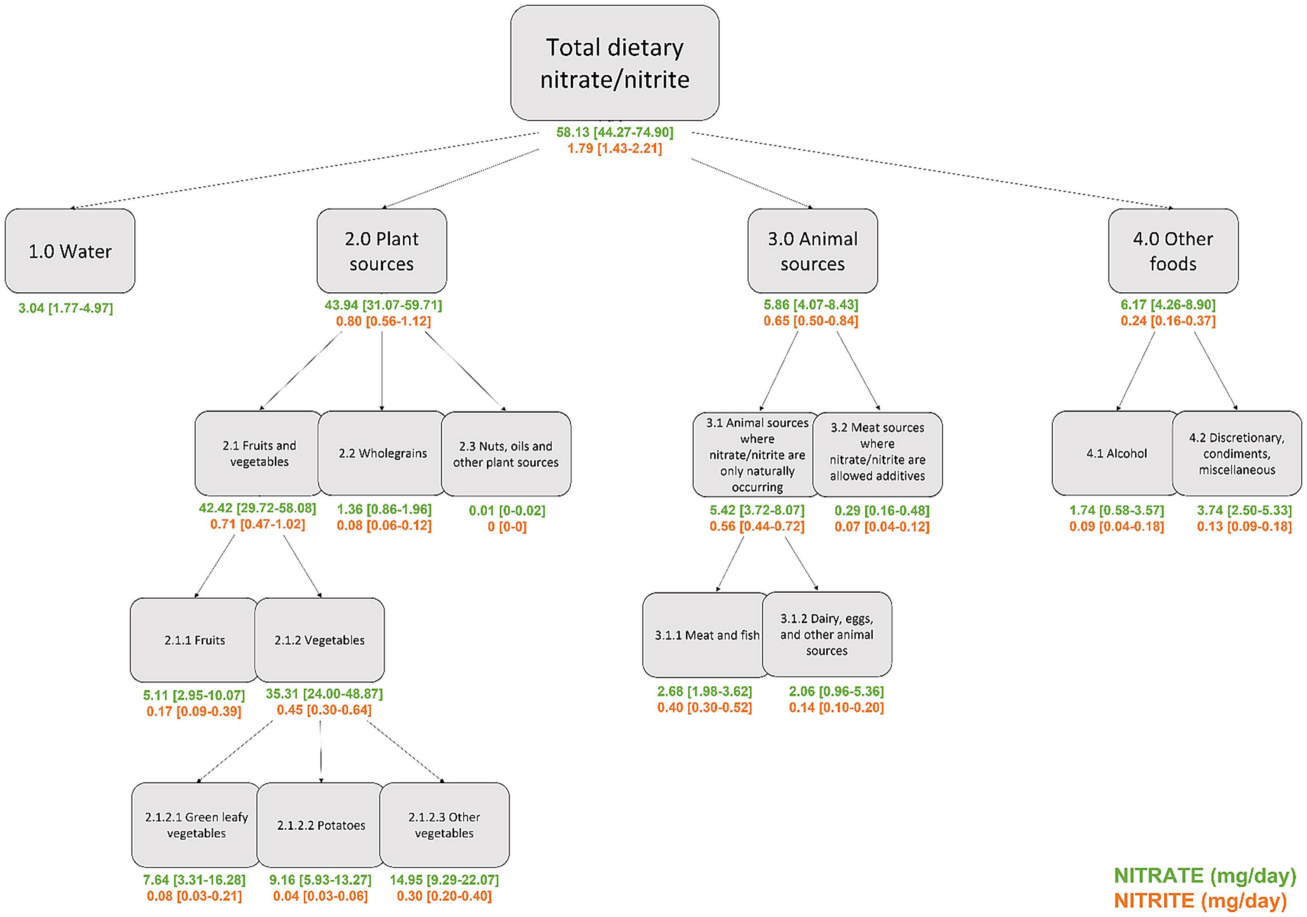
Intakes (median [IQR]) of nitrate and nitrite, grouped by source, in the Danish Diet Cancer and Health cohort (*n* = 55,754).

In a validated semi-quantitative 192-item food frequency questionnaire (FFQ) (22–24), cohort participants were asked to report their average intake of different food and beverage items, including tap water, over the past 12 months within 12 possible categories ranging from never to 8 times or more per day. Daily intake of each food/beverage was calculated for each participant using the software program FoodCalc, using standardized recipes and portion sizes specifically developed for this questionnaire. To enable source-specific analysis of nitrate and nitrite intake, all food and beverage items from the FFQ were grouped into four overarching categories: tap water, foods from plant sources (i.e., fruits, vegetables, legumes, wholegrains, nuts and oils), foods from animal sources (i.e., red meat, poultry, offal, dairy products, eggs, fish, and other seafood products, and meat sources where nitrate and nitrite are allowed additives), and other sources (i.e., alcoholic beverages and discretionary foods) (Figure 1). Sub-categories were made to investigate group-level contributors to nitrate/nitrite intake; further information on the assignment of food and beverage items from the FFQ to the overarching categories and each sub-category can be found in the Supplementary methods.

#### 2.2.1 Nitrate and nitrite from plant sources

Nitrate and nitrite intakes from plant sources at baseline were quantified using a comprehensive nitrate/nitrite database, which comprised more than 13,000 entries extracted from 396 publications [database version number: Veg2020V1.2052022 (20)] as well as 5,619 entries from analyses undertaken by governments as part of national monitoring programs. As the nitrate concentration of vegetables varies according to geographical location (25), to reflect the diet of the cohort participants, we used median nitrate values obtained from vegetables available for purchase in Denmark. If there were less than three references for nitrate values obtained in Denmark, the median value for Northern Europe was used. If there were less than three references for nitrate values obtained in Northern Europe, a median value of all European countries was used. If there were no nitrate values for a vegetable obtained in Europe, then the median value used considered references for all countries available in the dataset. An estimated 50% loss of nitrate content in raw vegetables was considered for boiled vegetables as boiling has been shown to approximately halve their nitrate content (20). After the median nitrate/nitrite values were assigned to each of the plant-sourced ingredients that made up each food item in the FFQ, nitrate/nitrite intake was calculated by multiplying the reported quantity of consumption for each ingredient (g/day) by its assigned median nitrate/nitrite value (mg/g). Total dietary nitrate intake from plant-based foods was then calculated as the sum of the nitrate content of each individual plant-sourced ingredient.

#### 2.2.2 Nitrate and nitrite from animal sources

For the estimation of nitrate and nitrite intakes from animal sources at baseline, the respective database comprised 6,245 entries for nitrate and 6,365 for nitrite, extracted from 211 publications [database version number: Animal2022v1.0 (6)], as well as 5,040 entries from analyses undertaken by governments as part of national monitoring programs. The same preference for geographically obtained nitrate and nitrite values described above, and therefore methodology to assign these, was used to quantify intakes of all animal-based ingredients. This was especially important considering Denmark has a lower threshold for permitted levels of nitrate and nitrite used as preservatives in processed meat products than those of the European Union and other countries (18). Total dietary nitrate/nitrite intake from animal-sourced ingredients were then calculated as the sum of the nitrate/nitrite content of each individual animal-sourced ingredient, respectively.

#### 2.2.3 Nitrate from tap water

Nitrate from tap water was assessed using the Danish public national geodatabase Jupiter, which includes multiple parameters regarding waterworks and tap water quality, including nitrate content, over decades (26, 27). Data were spatially linked with the addresses of the cohort participants and, using this geocoding approach, the nitrate content of tap water was estimated on an individual-level for each cohort participant. We used historical addresses of the cohort participants by tracing their residential histories in the civil registration system between 1978 and 2016. Each address was assigned an annual mean of the nitrate concentrations at the waterworks that supplied this address. For addresses that were supplied by more than one waterworks, a water-production-volume weighted annual mean was calculated, if volume information was available, otherwise, a simple mean was calculated. For years with no nitrate samples at water supply area-level, nitrate concentrations were imputed by linear regression between two sampled years and by last observation carried forward and next observation carried backward at the fringes. The number of years to the next available sampled year was calculated. While the limit of detection (LOD) varied over time, the maximum LOD was 1 mg/L. Each address within 100 m of a registered private drinking water well was classified as being supplied by a private well. Since nitrate samples in private wells are sparse, no time-series of nitrate levels were computed here, instead an all-time average nitrate level at the private well was assigned to the address. To estimate intakes of water-sourced nitrate (mg/d) at baseline, we first calculated intakes of tap water from the food frequency questionnaire as the sum of intakes of tap water, tea, coffee and water added to fruit syrup (L/d) for each participant. This value was multipled by the time-weighted average of the nitrate concentration (mg/L) at every address each cohort participant lived at in the 12 months prior to their enrolment into the study.

### 2.3 Sociodemographic information

Information on age, sex, smoking status (current, former, never), and education level (years of schooling) was obtained from self-administered questionnaires completed by participants upon enrolment. Alcohol consumption (g/d) was obtained from the FFQ. Physical activity was assessed by a questionnaire in which leisure time and transport-related activity was reported as hours/week spent on sports, cycling, gardening, walking, housework, and “do-it-yourself” activities and converted to a total daily metabolic equivalent of task (MET) score. Participant BMI was computed from anthropometric measurements taken by trained personnel at study centers at the time of enrolment.

### 2.4 Statistical analysis

Baseline characteristics are presented for the total cohort, as well as stratified by lowest (quintile 1) and highest (quintile 5) intakes of nitrate and nitrite from plant, animal, and water sources. As source-dependent nitrate/nitrite intakes, and intakes of the major contributing food groups were non-normally distributed, Spearman’s rank correlation coefficients (ρ) were calculated. Radar plots were drawn to visualize differences in underlying dietary patterns (percentage differences in intakes of 14 major food groups) between participants in the lowest and highest quintiles of nitrate intake from plant, animal and water sources. The median intake of the entire cohort was used as the reference category and food group intakes were adjusted for total energy intake before plotting. To explore predictors of source-dependent nitrate intake, we cross-sectionally examined associations between sociodemographic factors and nitrate intake in multivariable and mutually adjusted linear regression models. Nitrate intake from plant and animal sources are presented with and without adjustment for total energy intake. When nitrate intake from tap water was the outcome of interest, we further adjusted for total tap water consumption (g/day) as we hypothesized that total water consumption may be differentially associated with several sociodemographic factors. Due to multiple testing, only *p*-values<0.001 were considered statistically significant. Box plots were created to illustrate changes in tap water nitrate concentration over time in public wells supplying the addresses of the cohort participants. Owing to the high degree of skewness in the distribution, natural log values of the water nitrate concentration (mg/L) were used. All analyses were undertaken using Stata/IC 16.0 (StataCorp LLC) and R statistics (28).

## 3 Results

### 3.1 Overall and source-specific nitrate and nitrite intakes

In this cohort of 55,754 Danish citizens, aged 50–65 years, of which 53.3% were female, median [IQR] estimated intakes of dietary nitrate and nitrite were 58.13 [44.27–74.90] mg/d and 1.79 [1.43–2.21] mg/d, respectively. Focusing on nitrate intake, the largest contributor was plant-sourced foods (median [IQR]: 43.94 [31.07–59.71] mg/d; Figure 1). The top contributors to plant-sourced nitrate intake were potato (~25%), lettuce (~14%), carrot and banana (~8% each). There was a high correlation between intakes of nitrate from plant-sources and intakes of all plant-based foods themselves (*ρ* = 0.80, Supplementary Figure 2). Nitrate from animal sources made up approximately one tenth of estimated total nitrate intake (median [IQR]: 5.86 [4.07–8.43] mg/d). Only a small proportion of dietary nitrate came from meat sources where nitrate/nitrite are allowed additives (median [IQR]: 0.29 [0.16–0.48] mg/d). The top contributors to animal-sourced nitrate intake were beef (~29%) and yoghurt (~25%). There was a moderate correlation between intakes of nitrate from animal-sources and intakes of animal-based foods (*ρ* = 0.61) while intakes of nitrate from meat sources where nitrate/nitrite are allowed additives were very highly correlated with processed meat intake (*ρ* = 0.95, Supplementary Figure 3). The median [IQR] intake of nitrate intake from tap water was 3.04 [1.77–4.97] mg/d. Intakes of nitrate from tap water were not correlated with intakes of nitrate from any other source (Supplementary Figure 4). Intakes of dietary nitrite came predominantly from vegetables, meat and fish, and fruits, with only a very small proportion coming from meat sources where nitrate/nitrite are allowed additives (Figure 1). The top contributors to plant-sourced nitrite intake were lettuce (~13%), tomato (~12%), and apple (~8%) while the top contributors to animal-sourced nitrite intake were pork (~30%), beef (10%) and cheese (~8%). Although intakes of dietary nitrite were much lower than those of nitrate, when plants and animal products where nitrate/nitrite are allowed additives were the sources, intakes of these two compounds were highly correlated (*ρ* = 0.77 and 0.94, respectively), whereas intakes of nitrate and nitrite from all animal sources were only moderately correlated (*ρ* = 0.59, Supplementary Figure 4).

### 3.2 Baseline characteristics by source-specific nitrate and nitrite intakes

The baseline sociodemographic and dietary characteristics of cohort participants, overall and stratified by the lowest (quintile 1) and highest (quintile 5) intakes of the three primary dietary sources of nitrate, are presented in Table 1. Compared to participants with the lowest intakes of nitrate from plant sources, a higher proportion of those with the highest intakes were female, never smokers, with a high education level and they tended to be younger, more physically active, and have a lower BMI. For nitrate intake from animal sources, a higher proportion of participants with the highest intakes were male, former smokers, with a high education level and they tended to be more physically active and have a slightly higher BMI, when compared to those with the lowest intakes. A higher proportion of participants with the highest intakes of nitrate from tap water were female, current smokers, with a low education level and they tended to be more physically active than those with the lowest intakes. Similar patterns were observed for high versus low intakes of nitrite from plant and animal sources (Supplementary Table 1).

**Table 1:** Baseline characteristics of study population by quintiles of nitrate intake from the three dietary sources.

	Total population	Nitrate intake from plant sources		Nitrate intake from animal sources		Nitrate intake from drinking water	
	*N* = 55,754	Quintile 1 (*n* = 11,151)	Quintile 5 (*n* = 11,151)	Quintile 1 (*n* = 11,151)	Quintile 5 (*n* = 11,151)	Quintile 1 (*n* = 11,147)	Quintile 5 (*n* = 11,151)
Nitrate intake from plant sources (mg/day)	43.9 [31.1–59.7]	22.2 [18.1–25.4]	76.8 [69.8–88.3]	36.4 [24.6–52.4]	52.7 [39.1–69.5]	42.2 [30–57]	44.6 [31.3–61.4]
Nitrate intake from animal sources (mg/day)	5.9 [4.1–8.4]	4.6 [3.4–6.7]	7.1 [4.7–9.9]	3.0 [2.5–3.4]	11.2 [9.9–13.9]	5.8 [4.1–8.3]	5.9 [4.0–8.5]
Nitrate intake from drinking water (mg/day)	3.0 [1.8–5.0]	2.9 [1.7–4.9]	3.3 [2.0–5.2]	3.2 [1.9–5.2]	3.1 [1.8–5.0]	1 [0.7–1.3]	8.4 [6.7–12.4]
Demographics							
Age (years)							
50–54	23,543 (42.2)	4,405 (39.5)	4,944 (44.3)	4,601 (41.3)	4,501 (40.4)	4,903 (44.0)	4,810 (43.1)
55–60	20,241 (36.3)	4,175 (37.4)	3,986 (35.7)	4,120 (36.9)	4,075 (36.5)	3,936 (35.3)	4,107 (36.8)
61–65	11,970 (21.5)	2,571 (23.1)	2,221 (19.9)	2,430 (21.8)	2,575 (23.1)	2,308 (20.7)	2,234 (20.0)
Sex (male)	26,576 (47.7)	5,638 (50.6)	4,660 (41.8)	3,258 (29.2)	5,962 (53.5)	6,309 (56.6)	4,496 (40.3)
BMI (kg/m^2^)	25.5 [23.3–28.2]	26.0 [23.6–28.9]	25.0 [22.9–27.6]	25.1 [22.8–27.9]	25.6 [23.4–28.3]	25.7 [23.4–28.2]	25.6 [23.3–28.4]
MET score	56.5 [37.0–85.0]	48.5 [30.5–75.5]	64.5 [43.0–93.5]	53 [34.0–79.5]	61 [39.5–90.8]	52 [34.5–77.5]	60 [38.2–90.0]
Smoking status							
Never	19,563 (35.1)	3,092 (27.7)	4,404 (39.5)	3,969 (35.6)	4,077 (36.6)	4,272 (38.3)	3,593 (32.2)
Former	16,056 (28.8)	2,648 (23.7)	3,672 (32.9)	2,976 (26.7)	3,344 (30.0)	3,374 (30.3)	3,374 (30.3)
Current	20,135 (36.1)	5,411 (48.5)	3,075 (27.6)	4,206 (37.7)	3,730 (33.4)	3,501 (31.4)	4,554 (40.8)
Education level							
≤7 year	18,354 (32.9)	4,907 (44.0)	2,630 (23.6)	3,841 (34.4)	3,454 (31.0)	3,650 (32.7)	4,017 (36.0)
8–10 years	25,697 (46.1)	4,940 (44.3)	4,886 (43.8)	5,381 (48.3)	4,886 (43.8)	5,112 (45.9)	5,198 (46.6)
≥11 years	11,703 (21.0)	1,304 (11.7)	3,635 (32.6)	1,929 (17.3)	2,811 (25.2)	2,385 (21.4)	1,936 (17.4)
Dietary characteristics							
Energy (kcal/day)	2,272 [1,879 – 2,721]	1,948 [1,600 – 2,340]	2,576 [2,174 – 3,060]	1,797 [1,526 – 2,113]	2,698 [2,291 – 3,185]	2,280 [1,894 – 2,710]	2,269 [1,859 – 2,733]
Red meat (g/day)	81.4 [58.6–111.5]	74.8 [54.6–100.6]	81.5 [55.7–116.3]	56 [42.1–69.5]	103.8 [74.8–141.8]	87.3 [63.3–117.5]	77.5 [55.9–107.1]
Processed meat (g/day)	21.6 [12.2–35.5]	22.4 [12.9–36.7]	19 [9.6–33.0]	14.3 [7.9–23.0]	26.2 [15.4–42.6]	23.2 [13.7–37.3]	21.0 [11.5–35.0]
Fish (g/day)	38.2 [25.4–55.3]	28.8 [18.8–42.1]	47.4 [31.7–68.0]	28.6 [18.8–41.4]	47.9 [32.3–68.3]	37.2 [24.7–53.0]	38.4 [25.5–56.2]
Poultry (g/day)	17.9 [10.3–27.7]	12.5 [6.8–19.9]	22.8 [13.4–35.3]	13.1 [6.8–21.3]	21.2 [12.9–33.1]	17.8 [10.5–26.9]	17.9 [10.0–28.3]
Dairy (g/day)	293.2 [152.8–558.9]	251.2 [107.2–537.0]	342.7 [201.9–602.2]	128.9 [66.9–275.9]	526.7 [366.7–784.8]	297.7 [156.2–564.3]	292.8 [151.9–557.0]
Butter (g/day)	9.2 [1.0–20.1]	10.5 [2.2–19.8]	7.5 [0.4–20.4]	6.1 [0.4–16.1]	10.7 [1.2–22.0]	10.0 [1.1–21.0]	8.4 [0.8–19.5]
Vegetable oil (g/day)	4.7 [1.2–8.9]	1.3 [0.5–2.9]	9.0 [4.7–13.5]	2.0 [0.7–6.2]	5.2 [1.6–11.3]	4.4 [1.2–8.7]	4.6 [1.0–8.9]
Wholegrains (g/day)	39.0 [25.0–56.4]	29.9 [21.8–45.6]	45.8 [30.0–63.8]	30.3 [22.0–46.6]	45.8 [29.6–64.1]	38.5 [24.2–55.4]	39.5 [25.5–57.3]
Refined grains (g/day)	45.9 [29.4–72.4]	43.4 [26.1–80.9]	48.2 [31.4–71.0]	37.5 [22.9–56.6]	50.5 [33.0–78.3]	46.9 [29.8–76.7]	45.2 [28.9–70.8]
Fruits (g/day)	169.8 [92.9–279.8]	86.7 [39.3–152.5]	282.9 [177.6–433.2]	143.1 [70.0–249.4]	216.7 [129.8–344.4]	157.5 [85.5–263.2]	176.6 [97.6–296.4]
Green leafy vegetables (g/day)	7.8 [3.6–19.6]	2.2 [1.0–3.9]	24.2 [18.3–36.5]	5.6 [2.2–18.0]	12.3 [4.8–21.1]	7.7 [3.5–19.4]	7.3 [3.4–19.4]
Potatoes (g/day)	127.3 [81.6–184.2]	93.1 [62.8–137.3]	145.2 [98.0–217.1]	110.7 [68.6–153.5]	137.5 [90.8–201.3]	132.3 [88.7–190.3]	126.1 [77.6–184.3]
Other vegetables (g/day)	154.5 [101.5–221.0]	72.3 [51.0–96.7]	282.2 [228.3–349.0]	124.7 [74.6–192.2]	190.3 [133.7–262.3]	146.6 [95.7–206.5]	158.4 [103.9–232.9]
Alcohol (g/day)	12.9 [5.9–31.1]	12.1 [3.6–32.3]	12.7 [6.0–29.6]	10.9 [3.2–25.8]	13.5 [6.4–30.9]	14.2 [6.5–32.4]	11.5 [4.7–24.5]

Data expressed as median [IQR] or *n* (%).

BMI, body mass index; MET, metabolic equivalent.

### 3.3 Differences in underlying dietary patterns across source-specific nitrate intake quintiles

**Figure 2:**
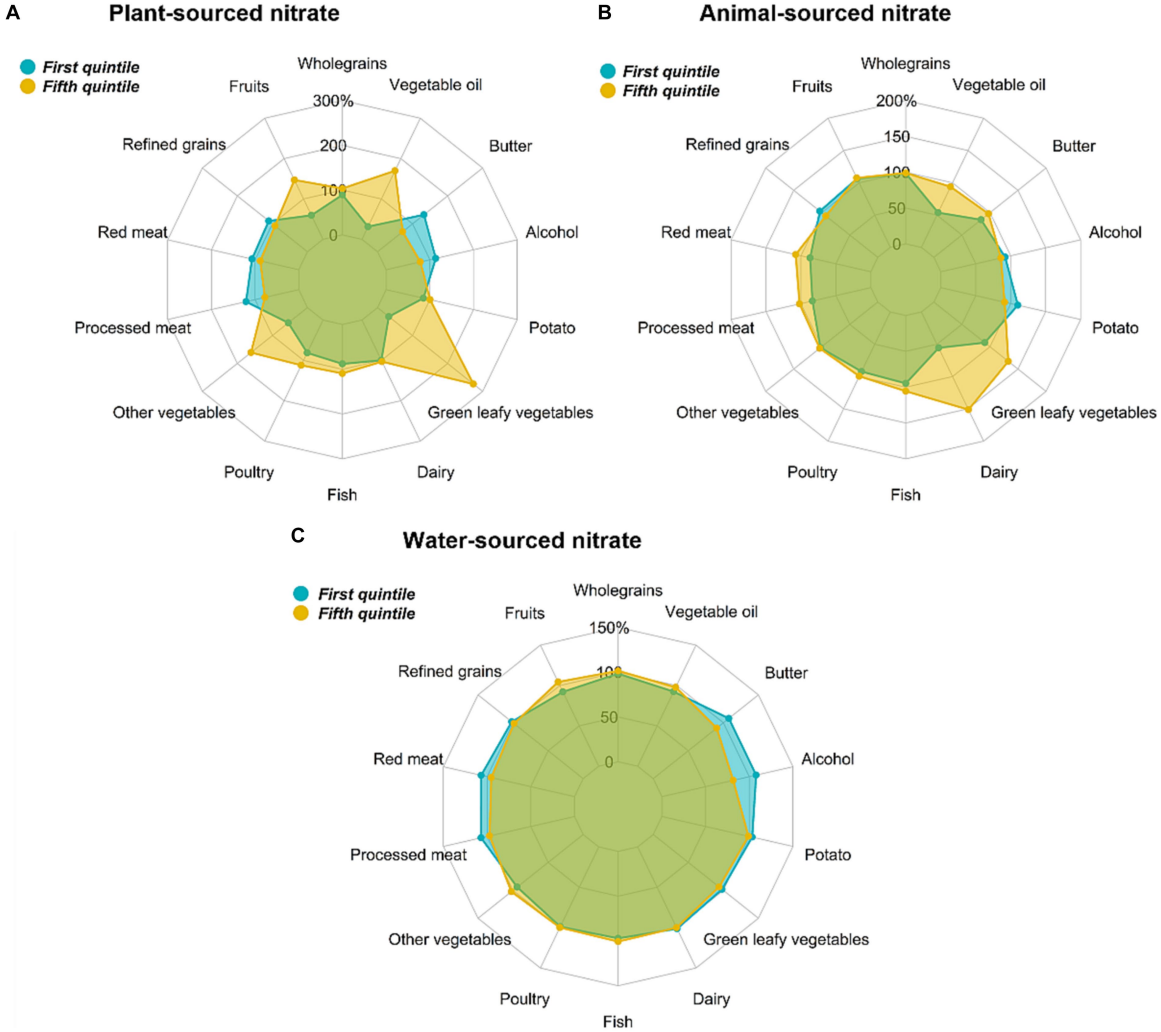
Radar plots depicting differences in energy-adjusted intakes of major food groups between participants with the highest (quintile 5) versus the lowest (quintile 1) intakes of **(A)** plant-sourced nitrate, **(B)** animal-sourced nitrate, and **(C)** water-sourced nitrate in participants of the Danish Diet Cancer and Health cohort. The percentages are relative to median intake of the entire cohort.

Participants with the highest intakes of nitrate from both plant and animal sources had a higher total energy intake than participants with the lowest nitrate intakes (Table 1). Intake levels of foods from the 14 different food groups were all higher in participants with the highest intakes of nitrate from plant and animal sources, except for processed meats and butter (higher intake among participants with the lowest intakes of plant-sourced nitrate). Overall energy intake did not differ between high and low consumers of nitrate from tap water, but compared to participants in the highest quintile, those in the lowest quintile consumed less fruit, and vegetables other than green leafy vegetables, and more red meat, potatoes, and alcohol (Table 1). After accounting for their higher energy intake, the diet of participants in the highest intake quintile of plant-sourced nitrate intake had higher proportions of vegetables (green leafy and other), fruits, poultry, fish and vegetable oil and lower proportions of red meat, processed meat, butter, refined grains, and alcohol compared to participants in the lowest plant-sourced nitrate intake quintile (Figure 2A). For the highest, compared to the lowest, consumers of animal-sourced nitrate, after accounting for their higher energy intake, green leafy vegetables, vegetable oils, dairy products and meat (red meat, processed meat, poultry and fish) made up a higher proportion of their diet (Figure 2B). The underlying dietary pattern was more consistent between the highest and the lowest consumers of nitrate from tap water, although higher intakes of red and processed meat, butter and alcohol were observed in participants with the lowest intakes of nitrate from tap water (Figure 2C).

### 3.4 Sociodemographic predictors of source-specific nitrate intake

Compared to participants aged 50–54 years at baseline, those aged 61–65 years had a 0.25 mg/d higher intake of animal-sourced nitrate and a 1.29 and 0.41 mg/d lower intake of plant-and water-sourced nitrate, respectively (Model 2; Table 2). Compared to females, males had a lower intake of nitrate from all three sources (plant: −10.02 mg/d; animal: −0.84 mg/d; water: −0.84 mg/d). Participants with obesity had a 2.62 mg/d lower intake of plant-sourced nitrate and a 0.30 mg/d and 0.28 mg/d higher intake of animal-and water-sourced nitrate, respectively. Physical activity was only a significant predictor of intakes of nitrate from plant-sources; participants with a MET score ≥ 73.75 had a 4.07 mg/d higher intake compared to those with a MET score ≤ 43.50. Compared to a low intake of alcohol (>0–1 units/day), a high alcohol intake (more than 2 units/day) was associated with lower nitrate intake from all three sources (plant: −3.93 mg/d; animal: −0.71 mg/d; water: −0.59 mg/d). Compared to participants who had never smoked, those who smoked currently had a 0.31 mg/d and a 0.28 mg/d lower nitrate intake from plant-and animal-sources, respectively and a 0.58 mg/d higher intake of nitrate from tap water. Finally, a high education level (≥11 years) was associated with a 10.71 mg/d and 0.46 mg/d higher intake of nitrate from plant-and animal-sources, respectively, and a 0.80 mg/d lower intake of nitrate from tap water, when compared to a lower education level (≤7 years).

**Table 2:** Predictors of nitrate intake by source.

		Plant-sourced nitrate				Animal-sourced nitrate				Water-sourced nitrate			
		Model 1		Model 2		Model 1		Model 2		Model 1		Model 2	
	*n*	β (95% CI)	*p*-val.	β (95% CI)	*p*-val.	β (95% CI)	*p*-val.	β (95% CI)	*p*-val.	β (95% CI)	*p*-val.	β (95% CI)	*p*-val.
Age (years)													
50–54	23,543	ref.		ref.		ref.		ref.		ref.		ref.	
55–60	20,241	−0.70 (−1.11, −0.30)	<0.001	−0.29 (−0.66, 0.08)	0.127	0.15 (0.08, 0.23)	<0.001	0.24 (0.17, 0.30)	<0.001	−0.15 (−0.28, −0.02)	0.028	−0.14 (−0.27, −0.01)	0.041
61–65	11,970	−1.43 (−1.90, −0.95)	<0.001	−1.29 (−1.72, −0.85)	<0.001	0.25 (0.16, 0.34)	<0.001	0.28 (0.20, 0.36)	<0.001	−0.41 (−0.56, −0.26)	<0.001	−0.41 (−0.56, −0.25)	<0.001
Sex													
Female	29,178	ref.		ref.		ref.		ref.		ref.		ref.	
Male	26,576	−2.24 (−2.63, −1.86)	<0.001	−10.02 (−10.40, −9.64)	<0.001	0.67 (0.60, 0.74)	<0.001	−0.84 (−0.91, −0.77)	<0.001	−0.65 (−0.77, −0.52)	<0.001	−0.84 (−0.98, −0.71)	<0.001
BMI (kg/m^2^)													
Underweight (BMI <18.5)	435	1.51 (−0.53, 3.55)	0.145	0.25 (−1.61,2.11)	0.791	0.28 (−0.09, 0.65)	0.134	0.04 (−0.30, 0.36)	0.841	−0.41 (−1.07, 0.25)	0.220	−0.44 (−1.10, 0.21)	0.188
Normal weight (BMI 18.5–24.9)	24,006	ref.		ref.		ref.		ref.		ref.		ref.	
Overweight (BMI 25.0–29.9)	23,231	−1.54 (−1.94, −1.15)	<0.001	−0.93 (−1.29, −0.57)	<0.001	0.08 (0.00, 0.15)	0.037	0.20 (0.13, 0.26)	<0.001	0.10 (−0.03,0.22)	0.144	0.11 (−0.02,0.24)	0.091
Obese (BMI ≥30)	8,082	−3.15 (−3.70, −2.59)	<0.001	−2.62 (−3.13, −2.12)	<0.001	0.20 (0.10, 0.30)	<0.001	0.30 (0.21, 0.39)	<0.001	0.27 (0.09, 0.45)	0.003	0.28 (0.11, 0.46)	0.002
Physical activity level (MET score)													
≤43.50	18,889	ref.		ref.		ref.		ref.		ref.		ref.	
43.75–73.50	18,481	4.01 (3.58, 4.45)	<0.001	2.55 (2.15, 2.95)	<0.001	0.31 (0.23, 0.39)	<0.001	0.03 (−0.05, 0.10)	0.477	0.04 (−0.10, 0.18)	0.584	0.00 (−0.14, 0.14)	0.954
≥73.75	18,384	7.39 (6.95, 7.83)	<0.001	4.07 (3.66, 4.47)	<0.001	0.69 (0.62, 0.77)	<0.001	0.05 (−0.03, 0.12)	0.216	0.25 (0.11,0.39)	<0.001	0.17 (0.02, 0.31)	0.021
Alcohol intake (units/day)*													
Abstainer	1,291	−0.30 (−1.50, 0.91)	0.630	−0.25 (−1.35, 0.84)	0.652	0.02 (−0.20, 0.24)	0.842	0.03 (−0.17, 0.22)	0.791	0.36 (−0.02, 0.75)	0.065	0.37 (−0.02, 0.75)	0.064
>0–1	24,700	ref.		ref.		ref.		ref.		ref.		ref.	
>1–2	13,217	0.54 (0.08, 1.01)	0.021	−0.33 (−0.75, 0.09)	0.126	0.12 (0.04, 0.20)	0.005	−0.05 (−0.12, 0.03)	0.194	−0.08 (−0.23,0.07)	0.270	−0.10 (−0.25,0.04)	0.168
>2	16,546	−0.85 (−1.30, −0.40)	<0.001	−3.93 (−4.34, −3.51)	<0.001	−0.11 (−0.19, −0.02)	0.011	−0.71 (−0.79, −0.64)	<0.001	−0.51 (−0.66, −0.37)	<0.001	−0.59 (−0.75, −0.44)	<0.001
Smoking status													
Never	19,563	ref.		ref.		ref.		ref.		ref.		ref.	
Former	16,056	1.00 (0.54, 1.45)	<0.001	1.43 (1.02, 1.84)	<0.001	−0.19 (−0.11, −0.28)	<0.001	−0.11 (−0.19, −0.04)	0.002	0.22 (0.07, 0.36)	0.004	0.22 (0.08, 0.37)	0.003
Current	20,135	−5.32 (−5.76, −4.89)	<0.001	−5.08 (−5.47, −4.68)	<0.001	−0.31 (−0.39, −0.23)	<0.001	−0.28 (−0.35, −0.21)	<0.001	0.60 (0.46, 0.74)	<0.001	0.58 (0.44, 0.72)	<0.001
Education level													
≤7 years	18,354	ref.		ref.		ref.		ref.		ref.		ref.	
8–10 years	25,697	3.45 (3.06, 3.89)	<0.001	3.84 (3.46, 4.22)	<0.001	0.06 (−0.01, 0.14)	0.098	0.14 (0.07, 0.20)	<0.001	−0.45 (−0.59, −0.32)	<0.001	−0.45 (−0.58, −0.31)	<0.001
≥11 years	11,703	11.35 (10.83, 11.87)	<0.001	10.71 (10.24, 11.18)	<0.001	0.57 (0.48, 0.67)	<0.001	0.46 (0.38, 0.55)	<0.001	−0.77 (−0.94, −0.60)	<0.001	−0.80 (−0.96, −0.63)	<0.001

Associations between selected demographic and lifestyle variables and nitrate intake from plant, animal, and water sources. Estimates [β (95% confidence intervals)] and *p*-values were obtained from separate linear regression models. Model 1 included all predictors of interest; Model 2 included all predictors plus energy intake when plant-sourced and animal-sourced nitrate intakes were the outcomes of interest and water intake when water-sourced nitrate intake was the outcome of interest. MET, metabolic equivalent. *1 unit = 12 g.

### 3.5 Trends in tap water nitrate concentration over time

**Figure 3:**
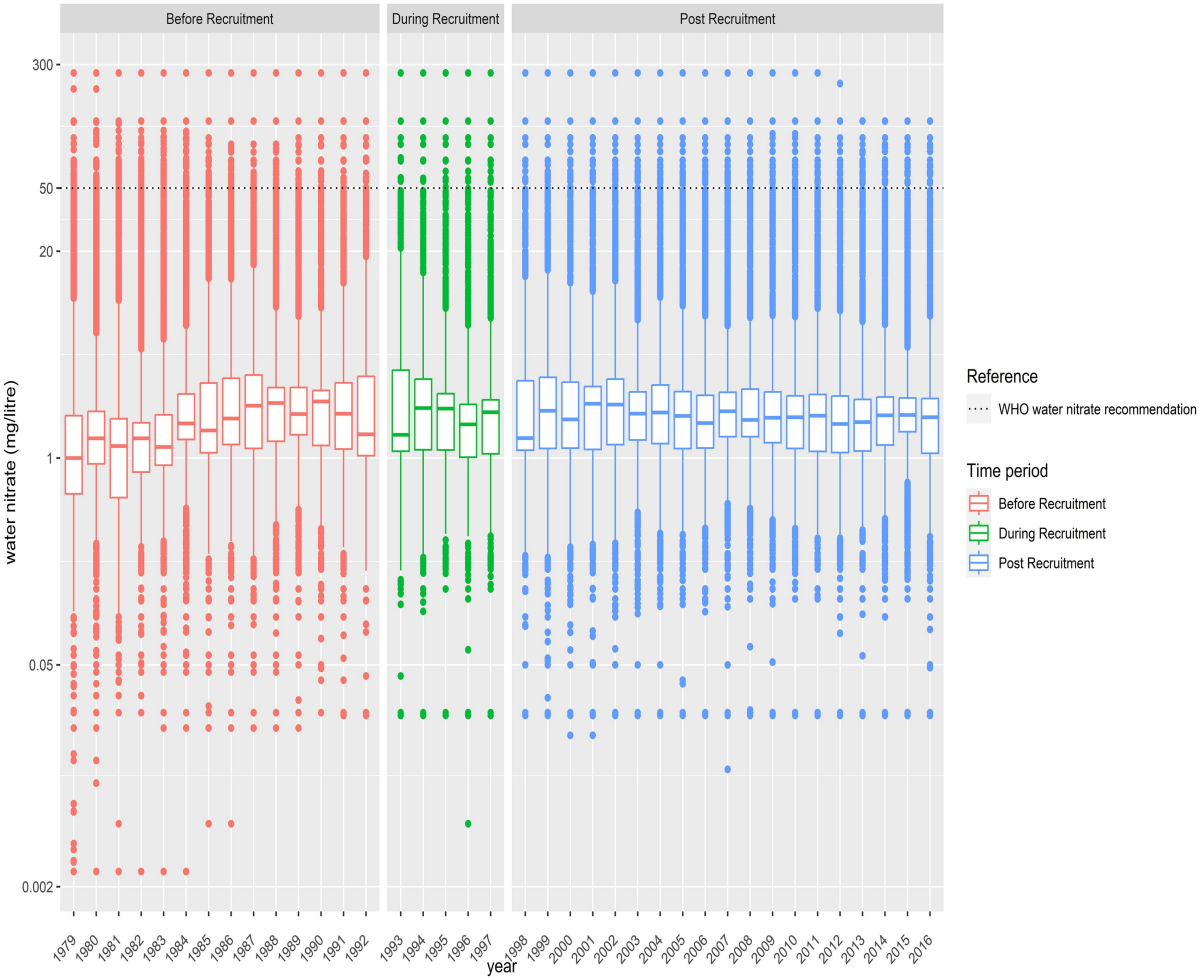
Box plots depicting the natural log-transformed concentration of nitrate (mg/L) reported in the public water supplies supplying participants of the Danish Diet Cancer and Health cohort with water from 1979 to 2016.

In the 12 months prior to enrolment (i.e., baseline), the concentration of nitrate in public waterworks supplying cohort participants ranged from below LOD to 42.1 mg/L (median [IQR]: 2.0 [1.2–2.8] mg/L) while the concentration of nitrate in private wells supplying cohort participants ranged from below the LOD to 265 mg/L (median [IQR]: 8.1 [1.1–40.0] mg/L). The median [IQR] intake of nitrate from tap water was 3.0 [1.8–4.9] mg/d for the 55,496 (99.5%) participants who received their tap water from a public supply and 15.1 [1.8–59.3] mg/d for the 258 (0.5%) participants who received their tap water from a private well. For public wells only, the distribution of water nitrate concentration was fairly constant from 1979 to 2016 (Figure 3). As the concentration of nitrate in public wells is interpolated from very few values, it is not possible to examine stability over time.

## 4 Discussion

In our cohort of 55,754 Danish citizens, plant-sourced foods contributed to 76% of nitrate intake, animal-based foods 10%, and water 5% with the remaining coming from other foods (i.e., alcohol, discretionary foods, condiments etc.). Nitrite intakes were more evenly sourced from both plants (45%) and animals (36%). Participants with a higher nitrate intake from plants typically exhibited healthier lifestyles and dietary habits. Conversely, those with higher nitrate intake from water showed more sociodemographic risk factors (i.e., smoking, obesity, lower education). The patterns for animal-sourced nitrate intake were less definitive.

The European Food Safety Authority (EFSA) has set the acceptable daily intake (ADI) of nitrate at 3.7 mg/kg of body weight per day, translating to ~260 mg/day for a 70 kg individual. In the present study, the median intake of dietary nitrate was ~58 mg/day, notably lower than the ADI and on the lower end of the International Agency for Research on Cancer’s (IARC) estimated global nitrate intake of 58–218 mg/day in 2010 (29). For nitrites, the ADI from EFSA is set at 0.07 mg/kg body weight/day. Our cohort’s median nitrite intake was 1.79 mg/day, significantly below the 4.9 mg/day ADI for a 70 kg individual but above IARC’s estimated global nitrate intake of 0.7–1.6 mg/day in 2010 (29). Prior estimates of nitrate intake in the Danish Diet Cancer and Health cohort were slightly higher (median [IQR] of 67 mg [36–105] (19)) than estimates in the present study, owing to slightly lower estimates of the nitrate content of key foods such as potatoes and broccoli in the new (20), compared to the original (25), nitrate database used. In our study, the primary dietary source of nitrate was vegetables (~61%), particularly potatoes, which, although they have a relatively low nitrate content (20), are consumed in large volumes, and lettuce. This is consistent with previous reports that potatoes and lettuce were the main dietary sources of nitrate in the Danish population between 1984 and 2011 (30). The primary dietary sources of nitrite in the studied cohort were vegetables, pork, and beef. Notably, less than 4% (median: 0.07 mg/d) of nitrite originated from meat sources where nitrate and nitrite additives are permitted. This is comparable to the approximately 4.7% nitrite contribution from food additives observed in the NutriNet-Santé cohort—a French online cohort beginning in 2009 (31)—though the actual intake was higher (mean: 0.3 mg/d). Investigating the relationship between nitrate/nitrite intake from meat sources where nitrate and nitrite additives are permitted and overall processed meat consumption can shed light on potential associations. Are they truly linked to nitrate/nitrite, or are these compounds merely indicative of greater processed meat consumption? In our study, the correlation was notably high (*ρ* = 0.95), and the correlation between nitrate and nitrite from allowable animal sources was also high (*ρ* = 0.94), suggesting that any associations derived from this data cannot be solely credited to nitrate or nitrite. This contrasts with the NutriNet-Santé cohort, where the correlation (Pearson’s) was only 0.24, and correlations between food additive nitrites/nitrates and foods containing processed meat were 0.73 and 0.67, respectively (31). Therefore, unlike the NutriNet-Santé cohort, our data does not allow for distinguishing between associations related to nitrate, nitrite, or processed meat consumption.

It is hypothesized that nitrate and nitrite, when consumed as part of plant-based whole foods, should not form carcinogenic *N*-nitroso compounds, largely due to the concurrent presence of vitamins and polyphenols in plants (16). Recently, very high levels of nitrite have been detected in samples of rucola, lettuce and spinach from Croatia (5); the safety of consuming vegetables with very high levels of nitrite, and nitrate, is currently unknown and warrants investigation. To better understand associations with health outcomes in future studies, it is essential to examine confounders of plant-sourced nitrate/nitrite intake, given its strong correlation with plant-based food consumption and the typically healthier behaviors of such consumers, including increased health consciousness and higher education levels (32). In the present cohort, physical activity and education level were positively, while age and BMI were inversely, independently associated with plant-sourced nitrate intake. Females and participants who had never smoked also had significantly higher plant-sourced nitrate intakes. Notably, participants with a higher intake of plant-sourced nitrate demonstrated a generally healthier underlying dietary pattern. Once energy intake was standardized, these individuals consumed more vegetables, fruits, poultry, fish, and vegetable oil and less red meat, processed meats, and alcohol than low plant-sourced nitrate consumers. This observed behavior is logically consistent with the premise that elevated exposure to plant-sourced compounds necessitates a higher consumption of plant foods, resulting in a lower meat intake when energy intake is held constant, although fish remains an exception. In guiding future research, it is pertinent to consider that adjusting for foods which contribute to the exposure of interest may not be methodologically sound. It is also worth noting that controlling for energy in such studies introduces a substitution bias (32) such that observed associations could either stem from higher intakes of the exposure (plant-sourced nitrate/nitrite in this instance) or the necessary lower intake of other food intakes (like meat and alcohol) required to preserve energy balance. Subsequent studies investigating the relationship between plant-sourced nitrate intake and health outcomes should judiciously control for factors like meat and alcohol consumption, especially if these can influence the outcome in question.

Owing to the presence of amines, amides or heme iron, and a lower and less diverse concentration of antioxidants than plant-based foods, nitrate/nitrite in animal-based foods may form *N*-nitroso compounds endogenously or exogenously (7). This has been demonstrated in the curing process of meat (8). While participants with a higher intake of plant-sourced nitrate typically had more favorable demographic, lifestyle, and dietary factors than low consumers, clear patterns were not so apparent between high versus low consumers of animal-sourced nitrate. In the present cohort, age, BMI, and education were positively and independently associated with animal-sourced nitrate intake while males and participants who smoked also had significantly higher animal-sourced nitrate intakes. After accounting for their higher total energy intake, participants with a higher nitrate intake from animal sources consumed more green leafy vegetables, vegetable oils and, of course, more dairy products and meat. Nitrate and nitrite occur naturally and are also used as additives, specifically as sodium and potassium nitrites (E249, E250) and nitrates (E251, E252), in animal-based foods (33). Their use as food preservatives is regulated by national and regional legislation (18, 34, 35) due to concerns that they may contribute to the adverse health effects associated with processed meat consumption. The potential differential biological effects of naturally occurring versus additive nitrate/nitrite remain unclear. However, recent observational studies are beginning to distinguish between these sources in their exposure definitions (31, 36). It might be prudent for future research to categorize by both the source (plant/animal/water) and the nature (naturally occurring versus additive) of nitrate and nitrite until more is known.

For over four decades, nitrate-contaminated drinking water and its potential association with cancer have raised public health concerns (37). Evidence suggests that consuming nitrate from drinking water can lead to endogenous *N*-nitrosamine formation in humans (38). Recent meta-analyses have linked drinking water nitrate with elevated risks of gastric and colorectal cancer, while some individual studies also hint at its association with other cancers (1). European Union guidelines, including those in Denmark, have set the maximum nitrate level in drinking water at 50 mg/L (39). The relevance of the present study is perhaps best appreciated in the context of earlier research. As an example, in a Danish nationwide population-based study using a historical longitudinal assessment of long-term drinking water nitrate exposure, the median nitrate concentration at the wells was 2.00 mg/L [IQR: 1.20, 2.85] (40) which closely matches the levels we report here. In that study, after controlling for age, sex, year of birth, previous cancer diagnosis and education, persons exposed to the highest level of drinking water nitrate had a 14% higher risk of colorectal cancer compared to those with the lowest exposure and risks were significantly higher at drinking water levels above 3.87 mg/L, well below the current drinking water standard. Notably, participants with elevated drinking water nitrate intake in our study generally had lower education, higher BMI, and a higher likelihood of smoking. If consistent with the population in the aforementioned study, these sociodemographic factors may have confounded observed associations as obesity and smoking are well-established risk factors for colorectal cancer (41). These factors should be accounted for in future studies where possible.

In this study, we aimed to describe intakes of source-dependent nitrate and nitrite and how they related to other demographic, lifestyle and dietary confounders. This groundwork will guide subsequent investigations into how source-specific nitrate and nitrite intakes relate to health outcomes. The study’s limitation is its reliance on one FFQ, which has potential inaccuracies due to its semi-quantitative design and fixed portion sizes, and that nitrate/nitrite intakes were only assessed at one time point. Moreover, the nitrate/nitrite estimates, derived from dietary questionnaires and food content databases, may not consider less common high-nitrate foods or account for variables like cultivation factors and postharvest conditions, which can influence nitrate levels, and thus cannot be interpreted as absolute intakes. Despite these challenges, the study boasts strengths including the utilization of the latest comprehensive databases on food and beverage nitrate content. Furthermore, it considers nitrate exposure from water, merging water intake data with longitudinal water nitrate levels specific to residency—a rarely seen combination offering a more precise insight into water nitrate exposure.

In this study, we have presented source-specific nitrate and nitrite intake levels in a cohort of 55,754 Danish citizens aged 50–65 years and described how differences in intake varied with underlying dietary patterns and important sociodemographic factors. The findings of this study will inform future studies aimed at investigating associations between source-specific nitrate/nitrite intakes and human health outcomes.

## Data availability statement

The datasets presented in this article are not readily available due to the sensitive nature of the data collected for this study. Requests to access the dataset from qualified researchers trained in human subject confidentiality protocols may be sent to the Diet Cancer and Health Steering Committee at the Danish Cancer Institute. Requests to access the datasets should be directed to dch@cancer.dk.

## Ethics statement

Ethical approval was not required for the studies involving humans because this study was done using previously collected data. The studies were conducted in accordance with the local legislation and institutional requirements. The participants provided their written informed consent to participate in this study.

## Author contributions

DE: Formal analysis, Writing – original draft. PP: Formal analysis, Writing – review & editing. CK: Funding acquisition, Supervision, Writing – review & editing. JS: Data curation, Funding acquisition, Methodology, Writing – review & editing. LZ: Data curation, Writing – review & editing. CB: Data curation, Funding acquisition, Writing – review & editing. FD: Funding acquisition, Writing – review & editing. PF: Project administration, Writing – review & editing. TS: Funding acquisition, Writing – review & editing. JH: Funding acquisition, Writing – review & editing. AO: Funding acquisition, Supervision, Writing – review & editing. AT: Funding acquisition, Supervision, Writing – review & editing. NB: Conceptualization, Data curation, Formal analysis, Funding acquisition, Investigation, Methodology, Project administration, Supervision, Writing – original draft, Writing – review & editing.

## References

[1] Bondonno CP Zhong L Bondonno NP Sim M Blekkenhorst LC Liu A. Nitrate: the Dr. Jekyll and Mr. Hyde of human health? Trends Food Sci Technol. (2023) 135:57–73. doi: 10.1016/j.tifs.2023.03.014

[2] Gupta KJ Mur LA Wany A Kumari A Fernie AR Ratcliffe RG. The role of nitrite and nitric oxide under low oxygen conditions in plants. New Phytol. (2020) 225:1143–51. doi: 10.1111/nph.15969, PMID: 31144317

[3] Dechorgnat J Nguyen CT Armengaud P Jossier M Diatloff E Filleur S. From the soil to the seeds: the long journey of nitrate in plants. J Exp Bot. (2011) 62:1349–59. doi: 10.1093/jxb/erq409 21193579

[4] Bian Z Wang Y Zhang X Li T Grundy S Yang Q. A review of environment effects on nitrate accumulation in leafy vegetables grown in controlled environments. Food Secur. (2020) 9:732. doi: 10.3390/foods9060732, PMID: 32503134 PMC7353485

[5] Luetic S Knezovic Z Jurcic K Majic Z Tripkovic K Sutlovic D. Leafy vegetable nitrite and nitrate content: potential health effects. Food Secur. (2023) 12:1655. doi: 10.3390/foods12081655, PMID: 37107450 PMC10137473

[6] Zhong L Liu AH Blekkenhorst LC Bondonno NP Sim M Woodman RJ. Development of a food composition database for assessing nitrate and nitrite intake from animal-based foods. Mol Nutr Food Res. (2021) 66:e2100272. doi: 10.1002/mnfr.202100272 34792849 PMC9540118

[7] Kuhnle G Bingham S. Dietary meat, endogenous nitrosation and colorectal cancer. Biochem Soc Trans. (2007) 35:1355–7. doi: 10.1042/BST0351355, PMID: 17956350

[8] Ozel MZ Gogus F Yagci S Hamilton JF Lewis AC. Determination of volatile nitrosamines in various meat products using comprehensive gas chromatography–nitrogen chemiluminescence detection. Food Chem Toxicol. (2010) 48:3268–73. doi: 10.1016/j.fct.2010.08.036, PMID: 20816717

[9] Iammarino M Berardi G Tomasevic I Nardelli V. Effect of different cooking treatments on the residual level of nitrite and nitrate in processed meat products and margin of safety (MoS) assessment. Food Secur. (2023) 12:869. doi: 10.3390/foods12040869, PMID: 36832944 PMC9956292

[10] WHO. Nitrate and nitrite in drinking-water: background document for development of WHO guidelines for drinking-water quality. (2003), Available at: https://apps.who.int/iris/handle/10665/75380.

[11] Petersson J Carlström M Schreiber O Phillipson M Christoffersson G Jägare A. Gastroprotective and blood pressure lowering effects of dietary nitrate are abolished by an antiseptic mouthwash. Free Radic Biol Med. (2009) 46:1068–75. doi: 10.1016/j.freeradbiomed.2009.01.011, PMID: 19439233

[12] Spiegelhalder B Eisenbrand G Preussmann R. Influence of dietary nitrate on nitrite content of human saliva: possible relevance to in vivo formation of N-nitroso compounds. Food Cosmet Toxicol. (1976) 14:545–8. doi: 10.1016/S0015-6264(76)80005-3, PMID: 1017769

[13] Tannenbaum S Weisman M Fett D. The effect of nitrate intake on nitrite formation in human saliva. Food Cosmet Toxicol. (1976) 14:549–52. doi: 10.1016/S0015-6264(76)80006-5 1017770

[14] Kobayashi J. Effect of diet and gut environment on the gastrointestinal formation of N-nitroso compounds: a review. Nitric Oxide. (2018) 73:66–73. doi: 10.1016/j.niox.2017.06.001, PMID: 28587887

[15] EFSA Panel on Contaminants in the Food Chain Schrenk D Bignami M Bodin L Chipman JK del Mazo J. Risk assessment of N-nitrosamines in food. EFSA J. (2023) 21:e07884. doi: 10.2903/j.efsa.2023.7884 36999063 PMC10043641

[16] Ahluwalia A Gladwin M Coleman GD Hord N Howard G Kim-Shapiro DB. Dietary nitrate and the epidemiology of cardiovascular disease: report from a National Heart, Lung, and Blood Institute workshop. J Am Heart Assoc. (2016) 5:e003402. doi: 10.1161/JAHA.116.003402, PMID: 27385425 PMC5015377

[17] IARC Working Group. Red Meat and Processed Meat: IARC Working Group on the Evaluation of Carcinogenic Risks to Humans. International Agency for Research on Cancer. (2015). 29949327

[18] COMMISSION REGULATION (EU) 2023/2108 of 6 October 2023 amending annex II to Regulation (EC) no 1333/2008 of the European Parliament and of the council and the annex to Commission Regulation (EU) no 231/2012 as regards food additives nitrites (E249-250) and nitrates (E 251–252). Official J Eur Union; (2023). Available at: http://data.europa.eu/eli/reg/2023/2108/oj (Accessed January 8, 2024).

[19] Bondonno CP Dalgaard F Blekkenhorst LC Murray K Lewis JR Croft KD. Vegetable nitrate intake, blood pressure and incident cardiovascular disease: Danish diet, Cancer, and health study. Eur J Epidemiol. (2021) 36:813–25. doi: 10.1007/s10654-021-00747-3, PMID: 33884541 PMC8416839

[20] Zhong L Blekkenhorst LC Bondonno NP Sim M Woodman RJ Croft KD. A food composition database for assessing nitrate intake from plant-based foods. Food Chem. (2022) 394:133411. doi: 10.1016/j.foodchem.2022.133411, PMID: 35753259

[21] Tjonneland A Olsen A Boll K Stripp C Christensen J Engholm G. Study design, exposure variables, and socioeconomic determinants of participation in diet, Cancer and health: a population-based prospective cohort study of 57,053 men and women in Denmark. Scand J Public Health. (2007) 35:432–41. doi: 10.1080/14034940601047986, PMID: 17786808

[22] Overvad K Tjoenneland A Haraldsdottir J Ewertz M Jensen OM. Development of a semiquantitative food frequency questionnaire to assess food, energy and nutrient intake in Denmark. Int J Epidemiol. (1991) 20:900–5. doi: 10.1093/ije/20.4.900, PMID: 1800428

[23] Tjoenneland A Overvad K Haraldsdottir J Bang S Ewertz M Jensen OM. Validation of a semiquantitative food frequency questionnaire developed in Denmark. Int J Epidemiol. (1991) 20:906–12. doi: 10.1093/ije/20.4.906, PMID: 1800429

[24] Tjoenneland A Haraldsdottir J Overvad K Stripp C Ewertz M Jensen OM. Influence of individually estimated portion size data on the validity of a Semiquantitative food frequency questionnaire. Int J Epidemiol. (1992) 21:770–7. doi: 10.1093/ije/21.4.770, PMID: 1521982

[25] Blekkenhorst LC Prince RL Ward NC Croft KD Lewis JR Devine A. Development of a reference database for assessing dietary nitrate in vegetables. Mol Nutr Food Res. (2017) 61:1600982. doi: 10.1002/mnfr.201600982, PMID: 28105786

[26] Schullehner J. Danish water supply areas and their links to water production facilities: an open-access data set. GEUS. Bulletin. (2022) 49:49. doi: 10.34194/geusb.v49.8319

[27] Schullehner J Stayner L Hansen B. Nitrate, nitrite, and ammonium variability in drinking water distribution systems. Int J Environ Res Public Health. (2017) 14:276. doi: 10.3390/ijerph14030276, PMID: 28282914 PMC5369112

[28] R Core Team. R: A language and environment for statistical computing. R Foundation for Statistical Computing, Vienna, Austria. (2022), Available at: https://www.R-project.org/

[29] IARC monographs on the evaluation of carcinogenic risks to humans. IARC monographs on the evaluation of carcinogenic risks to humans. Ingested nitrate and nitrite, and cyanobacterial peptide toxins, vol. 94. France: International Agency for Research on Cancer (2010). PMC4781178 21141240

[30] National Food Institute. Chemical contaminants 2004–2011. Soeborg: Technical University of Denmark (2015).

[31] Chazelas E Pierre F Druesne-Pecollo N Esseddik Y Szabo de Edelenyi F Agaesse C. Nitrites and nitrates from food additives and natural sources and cancer risk: results from the NutriNet-Santé cohort. Int J Epidemiol. (2022) 51:1106–19. doi: 10.1093/ije/dyac046, PMID: 35303088 PMC9365633

[32] Eakman T Metallinos-Katsaras E. What are the predictors, motivators, and barriers to reducing meat and following a more plant-based diet? Curr Dev Nutr. (2022) 6:481. doi: 10.1093/cdn/nzac059.009

[33] Honikel K-O. The use and control of nitrate and nitrite for the processing of meat products. Meat Sci. (2008) 78:68–76. doi: 10.1016/j.meatsci.2007.05.030 22062097

[34] EFSA Panel on Food Additives and Nutrient Sources added to Food Mortensen A Aguilar F Crebelli R Di Domenico A Dusemund B. Re-evaluation of sodium nitrate (E 251) and potassium nitrate (E 252) as food additives. EFSA J. (2017) 15:e04787. doi: 10.2903/j.efsa.2017.4787, 32625505 PMC7010087

[35] EFSA Panel on Food Additives and Nutrient Sources added to Food Mortensen A Aguilar F Crebelli R Di Domenico A Dusemund B. Re-evaluation of potassium nitrite (E 249) and sodium nitrite (E 250) as food additives. EFSA J. (2017) 15:e04786. doi: 10.2903/j.efsa.2017.4786, 32625504 PMC7009987

[36] Srour B Chazelas E Druesne-Pecollo N Esseddik Y de Edelenyi FS Agaësse C. Dietary exposure to nitrites and nitrates in association with type 2 diabetes risk: results from the NutriNet-Santé population-based cohort study. PLoS Med. (2023) 20:e1004149. doi: 10.1371/journal.pmed.1004149, PMID: 36649248 PMC9844911

[37] Hill M Hawksworth G Tattersall G. Bacteria, nitrosamines and cancer of the stomach. Br J Cancer. (1973) 28:562–7. doi: 10.1038/bjc.1973.186, PMID: 4593224 PMC2008943

[38] van Breda SG Mathijs K Sági-Kiss V Kuhnle GG Van der Veer B Jones RR. Impact of high drinking water nitrate levels on the endogenous formation of apparent N-nitroso compounds in combination with meat intake in healthy volunteers. Environ Health. (2019) 18:1–12. doi: 10.1186/s12940-019-0525-z 31623611 PMC6796425

[39] Union E. Council directive 91/676/EEC of 12 December 1991 concerning the protection of waters against pollution caused by nitrates from agricultural sources. Off J Eur Communities. (1991) 375:1–8.

[40] Schullehner J Hansen B Thygesen M Pedersen CB Sigsgaard T. Nitrate in drinking water and colorectal cancer risk: a nationwide population-based cohort study. Int J Cancer. (2018) 143:73–9. doi: 10.1002/ijc.31306, PMID: 29435982

[41] Sawicki T Ruszkowska M Danielewicz A Niedźwiedzka E Arłukowicz T Przybyłowicz KE. A review of colorectal cancer in terms of epidemiology, risk factors, development, symptoms and diagnosis. Cancers. (2021) 13:2025. doi: 10.3390/cancers13092025, PMID: 33922197 PMC8122718

