# Supplementary material for: Source-specific nitrate and nitrite intakes and associations with sociodemographic factors in the Danish Diet Cancer and Health cohort
Source: Front Nutr. 2024 Feb 27;11:1326991. doi: 10.3389/fnut.2024.1326991 (PMC10927827; doi:10.3389/fnut.2024.1326991)
Supplement: Supplementary file 1 [file Data_Sheet_1.docx]

Supplementary Material

# Supplementary Methods

**Total dietary nitrate**: the sum of the nitrate/nitrite content of all foods and beverages captured with the FFQ.

**1.0 Water**: the sum of the nitrate content in tap water and beverages where tap water was likely used (e.g., tea and coffee)

**2.0 Plant sources**: the sum of the nitrate/nitrite content of all plant-based foods including fruits, vegetables, wholegrains, nuts and plant oils.

The purpose of this group is to look at nitrate from plant-based foods which also contain vitamins and polyphenols in the whole food matrix.

**2.1 Fruits and vegetables**: the sum of the nitrate/nitrite content of all fruits and vegetables as defined in 2.1.1 and 2.1.2.

**2.1.1 Fruits**: the sum of the nitrate/nitrite content of all fruits.

NOTE: this group includes fruit juices and food items that in botanical terminology are vegetables but treated culturally as fruits (e.g., rhubarb).

**2.1.2 Vegetables**: the sum of the nitrate/nitrite content of all vegetables and legumes.

NOTE: this group includes vegetable juices and food items that in botanical terminology are fruits but treated culturally as vegetables (e.g., tomato).

**2.1.2.1 Green leafy vegetables**: the sum of the nitrate/nitrite content of all green leafy vegetables.

The purpose of this group is to look separately at the group of vegetables with the highest nitrate content.

**2.1.2.2 Potatoes**: the sum of the nitrate/nitrite content of potatoes. The purpose of this group is to look separately at potatoes as they have a relatively low nitrate content but are a major contributor to nitrate intake because of the volume consumed.

**2.1.2.3 Other vegetables**: the sum of the nitrate/nitrite content of all other vegetables, which do not fall into category 2.1.2.1 or 2.1.2.2.

**2.2 Wholegrains**: the sum of the nitrate/nitrite content of all wholegrain foods.

NOTE: this group includes brown breads, wholegrain cereals, brown rice etc., but does not include refined grains such as white bread, white rice, wheat flour etc.

The purpose of this group is to look at nitrate from grains which would also contain vitamins and polyphenols in the whole food matrix.

**2.3 Nuts, oils and other plant sources:** the sum of the nitrate/nitrite content of other foods from plant sources such as nuts and oils.

**3.0 Animal sources:** the sum of the nitrate/nitrite content of all animal-based foods including red meat, poultry, processed meat, offal, dairy products, eggs, fish and other seafood products.

**3.1** **Animal sources where nitrate/nitrite are only naturally occurring:** the sum of the nitrate/nitrite content of all animal-based foods where nitrate/nitrite are not allowed additives, as defined in 3.1.1 and 3.1.2.

**3.2 Meat sources where nitrate/nitrite are allowed additives**: the sum of the nitrate/nitrite content of all meats where nitrate and nitrite are allowed to be added.

The purpose of this group is to capture processed meats (red meats, poultry, pork, game) preserved with nitrate/nitrite. This category includes liver paste but does not include processed seafood and fish products (e.g., marinated herring, smoked salmon etc.) as the addition of nitrate/nitrite as a preservative is not permitted for these foods.

**3.1.1 Meat and fish**: the sum of the nitrate/nitrite content of all fresh red meat, poultry, fish, and other seafood products.

The purpose of this group is to capture meats and fish with only naturally occurring nitrate/nitrite. As such, this group includes fresh industrial processed meat products (including fresh sausages) that are industry-prepared and in which other food ingredients (e.g., common salt or spices) may have been added but which have not undergone curing.

**3.1.2 Dairy, eggs, and other animal sources:** the sum of the nitrate/nitrite content of all other foods of animal origin, which do not fall into category 3.1.1 or 3.2.

NOTE: this group includes cheeses, 100% butter and fermented dairy products, but does not include ice creams.

**4.0 Other foods**: the sum of the nitrate/nitrite content of all other foods and beverages, which do not fall into category 1.0, 2.0 or 3.0.

**4.1 Alcohol**: the sum of the nitrate/nitrite content of all alcoholic beverages.

**4.2 Discretionary foods, condiments and miscellaneous**: the sum of the nitrate/nitrite content of all other foods and beverages, which do not fall into category 1.0, 2.0 or 3.0, and are not alcohol.

NOTE: this group includes confectionary, savory snacks, refined grains (e.g., white rice, pasta, and processed cereals), soft drinks, ice cream, condiments, and mixed spreadable fats.

The purpose of this group is to capture unhealthy foods and foods which likely do not contain vitamins or polyphenols.

# Supplementary Figures


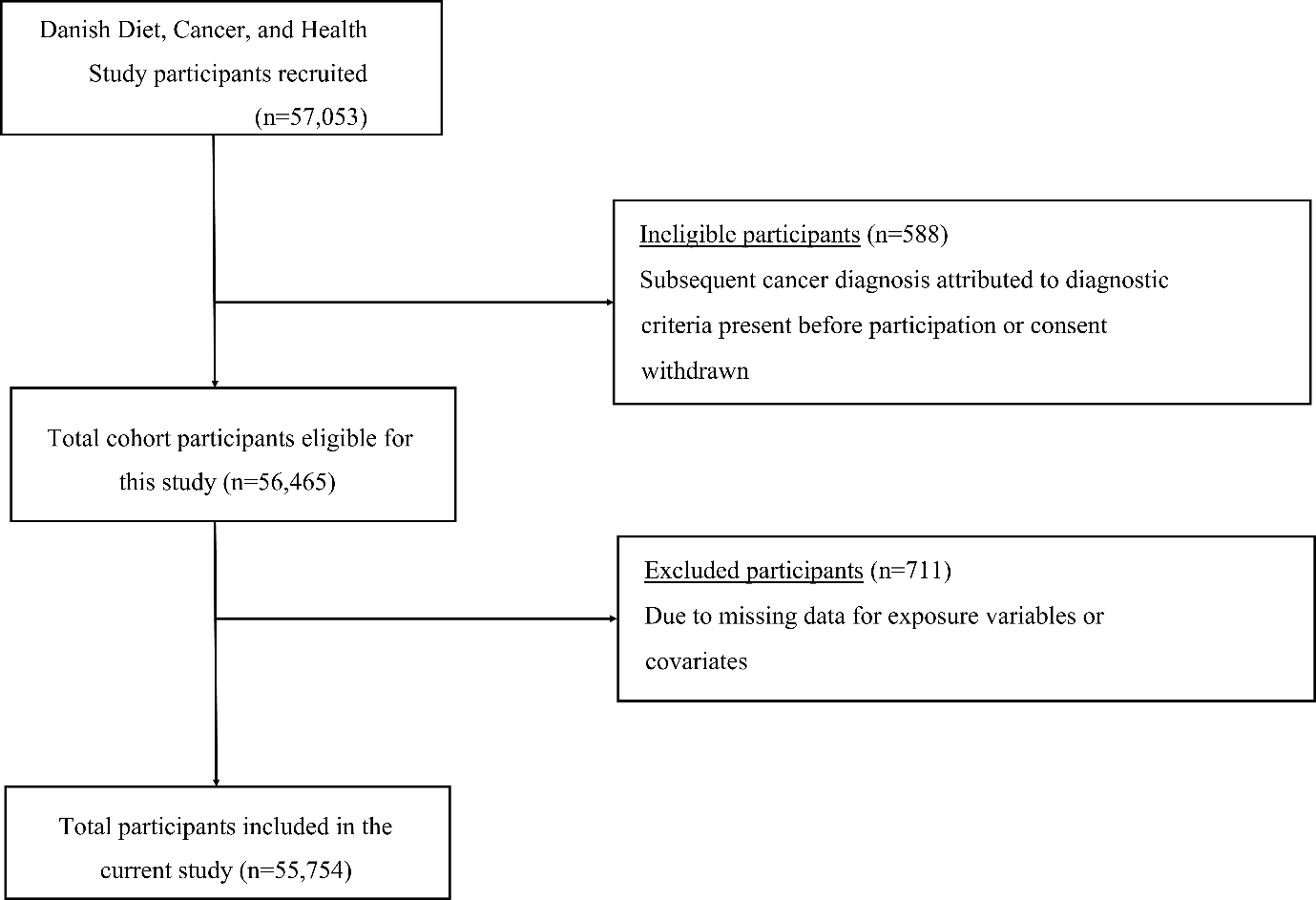


**Supplementary Figure 1.** Participant flowchart


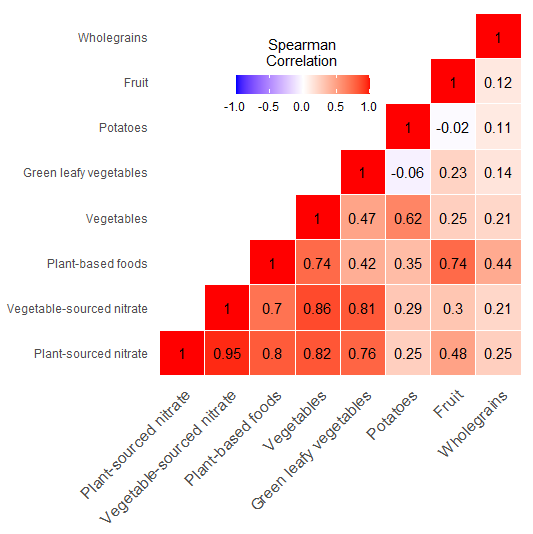


**Supplementary Figure 2.** Heat map depicting Spearman’s correlation coefficients between plant- and vegetable-sourced nitrate intake and the primary food groups contributing to plant-sourced nitrate intake in participants of the Danish Diet Cancer and Health cohort (p<0.001 for all).

**
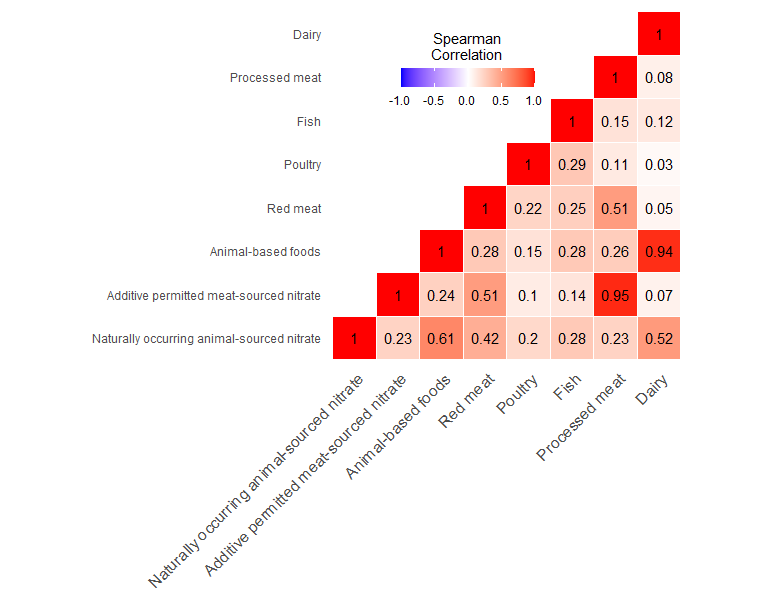
**

**Supplementary Figure 3.** Heat map depicting Spearman’s correlation coefficients between animal- and processed meat-sourced nitrate intake and the primary food groups contributing to animal-sourced nitrate intake in participants of the Danish Diet Cancer and Health cohort (p<0.001 for all).


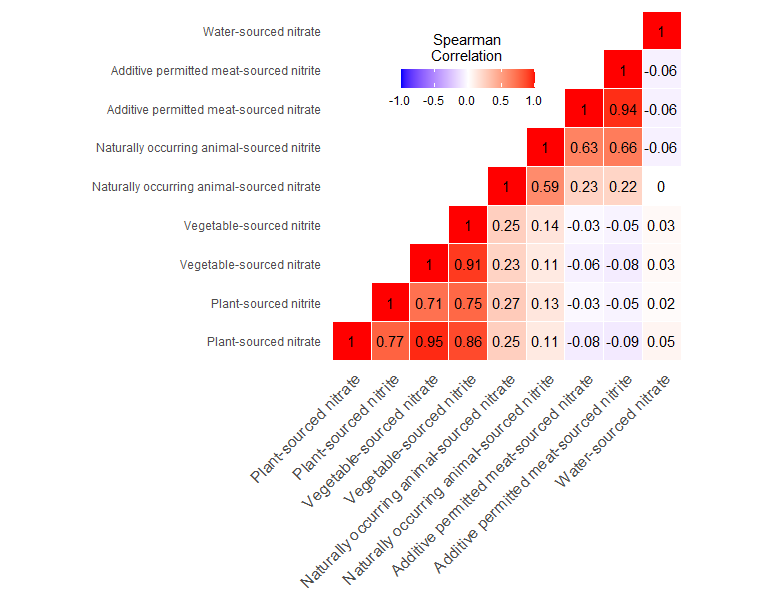


**Supplementary Figure 4.** Heat map depicting Spearman’s correlation coefficients between the different sources of dietary nitrate/nitrite in participants of the Danish Diet Cancer and Health cohort (p<0.001 for all).

| Supplementary Tables | | | |  |
| --- | --- | --- | --- | --- |
| **Supplementary Table 1. Baseline characteristics of study population by quintiles of nitrite intake from the plant and animal sources** | | | | |
|  | Nitrite intake from plant sources | | Nitrite intake from animal sources | |
|  | Quintile 1  (n=11,151) | Quintile 5  (n=11,151) | Quintile 1  (n=11,150) | Quintile 5  (n=11,151) |
| Nitrite intake from plant sources (mg/day) | 0.4 [0.3, 0.5] | 1.5 [1.3, 1.7] | 0.7 [0.5, 1.1] | 0.9 [0.6, 1.2] |
| Nitrite intake from animal sources (mg/day) | 0.6 [0.5, 0.8] | 0.7 [0.5, 0.9] | 0.4 [0.3, 0.4] | 1.1 [1.0, 1.2] |
| *Age (years)* |  |  |  |  |
| 50-54 | 4,220 (37.8) | 4,814 (43.2) | 4,809 (43.1) | 4,750 (42.6) |
| 55-60 | 4,171 (37.4) | 3,984 (35.7) | 4,160 (37.3) | 3,938 (35.3) |
| 61-65 | 2,760 (24.8) | 2,353 (21.1) | 2,181 (19.6) | 2,463 (22.1) |
| Sex (male) | 5,618 (50.4) | 4,961 (44.5) | 2,067 (18.5) | 8,348 (74.9) |
| BMI (kg/m^2^) | 26 [23.5, 28.8] | 25.1 [23.0, 27.6] | 24.6 [22.5, 27.2] | 26.3 [24.0, 29.1] |
| MET score | 49.5 [30.8, 77.5] | 63 [42, 93] | 54 [36.0, 79.5] | 60.5 [37.8, 92.5] |
| *Smoking status* |  |  |  |  |
| Never | 2,977 (26.7) | 4,414 (39.6) | 4,552 (40.8) | 3,150 (28.2) |
| Former | 2,582 (23.2) | 3,542 (31.8) | 3,121 (28.0) | 3226 (28.9) |
| Current | 5,592 (50.1) | 3,195 (28.7) | 3,477 (31.2) | 4775 (42.8) |
| *Education level* |  |  |  |  |
| ≤7 year | 5,070 (45.5) | 2,682 (24.1) | 2,891 (25.9) | 4,610 (41.3) |
| 8-10 years | 4,871 (43.7) | 4,990 (44.7) | 5,465 (49.0) | 4,589 (41.2) |
| ≥11 years | 1,210 (10.9) | 3,479 (31.2) | 2,794 (25.1) | 1,952 (17.5) |
| Energy (kcal/day) | 1,942.4 [1,596.1, 2,344.4] | 2,576.4 [2,181.2, 3,044.1] | 1,740.6 [1,476.0, 2,034.4] | 2,948.9 [2,556.8, 3,396.1] |
| Red meat (g/day) | 74.5 [ 54.3, 100.2] | 85.1 [ 59.4, 118.9] | 47.1 [35.3, 58.8] | 138.2 [109.3, 169.5] |
| Processed meat (g/day) | 22.1 [12.7, 36.0] | 20.7 [10.8, 34.6] | 9.5 [ 5.5, 15.3] | 42.3 [28.1, 61.4] |
| Fish (g/day) | 29.5 [19.5, 43.0] | 46 [30.7, 66.0] | 27.1 [17.9, 38.4] | 52.8 [35.2, 75.0] |
| Poultry (g/day) | 12.4 [ 6.8, 19.7] | 21.8 [13.0, 34.7] | 12.4 [ 6.6, 20.5] | 23.6 [14.4, 35.1] |
| Dairy (g/day) | 252.7 [106.9, 542.0] | 338.2 [203.9, 593.4] | 219.2 [ 89.3, 323.8] | 479.1 [256.0, 736.9] |
| Wholegrain (g/day) | 28.6 [21.6, 42.8] | 45 [29.4, 63.2] | 31.6 [22.2, 49.8] | 44.3 [29.5, 61.9] |
| Fruits (g/day) | 71.5 [ 34.2, 130.5] | 323.5 [225.7, 465.7] | 168.2 [ 89.6, 278.2] | 176 [ 95.1, 292.3] |
| Green leafy vegetables (g/day) | 2.4 [1.0, 4.2] | 20.4 [11.4, 32.8] | 7.8 [ 3.4, 19.6] | 7.3 [ 3.4, 19.4] |
| Potatoes (g/day) | 120.9 [ 72.4, 173.2] | 133.3 [ 89.2, 193.0] | 89 [ 60.8, 136.2] | 165.4 [116.7, 239.5] |
| Other vegetables (g/day) | 74.3 [52.3, 98.8] | 251.6 [183.7, 328.3] | 146.7 [ 90.6, 220.8] | 169.8 [115.7, 240.8] |
| Alcohol (g/day) | 12.1 [ 3.5, 32.2] | 13.2 [ 6.3, 30.5] | 10.6 [ 3.3, 22.8] | 15.6 [ 7.0, 35.9] |
| Data expressed as median [IQR] or n (%)  BMI, body mass index; MET, metabolic equivalent (determined from physical activity questionnaire) | | | |  |
